# Evaluation the gait of a subject with scoliosis while walking with and without Milwaukee orthosis

**DOI:** 10.1186/1748-7161-9-S1-P9

**Published:** 2014-12-04

**Authors:** Karimi Mohammad Taghi, Kavyani Mahsa, Reza M Mohammad Etemadifar

**Affiliations:** 1 Musculoskeletal Research Center, Isfahan University of Medical sciences, Isfahan, Iran; 2 Orthopedic Surgery Department, School of Medicine, Isfahan University of Medical sciences, Isfahan, Iran

**Keywords:** Orthosis, tendon length, gait, scoliosis

## Background

Scoliosis is one of the musculoskeletal disorders which influence the performance of the subjects during standing and walking. There is not enough information regarding the influence of orthosis on the gait and stability functions of scoliotic subjects, therefore the aim of this study is to find the effects of orthosis on the mentioned parameters.

## Case description

A 12 year old scoliotic subject was recruited in this study. They walked and stood with and without Milwaukee orthosis. Motions of the joint and the forces applied on the leg were collected by use of motion analysis system and a Kistler faceplate. The length of erector spine, external oblique and internal oblique abdominalis tendons was evaluated during walking with and without the orthosis by use of Open SIMM software.

## Results

The orthosis seems to improve the performance of the subjects during standing and walking. Moreover, it stretches the contracted muscles of vertebral column.

## Discussion

The orthosis aligned the vertebral column and improved the abilities of the subject to stand and walk. As the length of vertebral column muscles increased follow the use of the orthosis it can be concluded that it influenced the curve correction.

